# How Have Entrustable Professional Activities (EPAs) Been Implemented in Pharmacy Education? A Scoping Review

**DOI:** 10.3390/pharmacy13060156

**Published:** 2025-11-01

**Authors:** Luiz Claudio Oliveira Alves de Souza, Luciana Flavia de Almeida Romani, Marina Guimaraes Lima

**Affiliations:** 1 Post Graduation Programme in Medicines and Pharmaceutical Services, Federal University of Minas Gerais, Belo Horizonte 31270-901, Brazil; luigti@gmail.com (L.C.O.A.d.S.); lucianafalmeida@yahoo.com.br (L.F.d.A.R.); 2 Department of Social Pharmacy, Faculty of Pharmacy, Federal University of Minas Gerais, Belo Horizonte 31270-901, Brazil

**Keywords:** entrustable professional activities, pharmaceutical education, experiential learning, implementation science

## Abstract

Entrustable Professional Activities (EPAs) are units of professional practice entrusted to learners once they have attained the required competencies to perform them. This scoping review described how Entrustable Professional Activities (EPAs) have been implemented in pharmacy education. MEDLINE (PubMed), Scopus, and Google Scholar were searched (on 8 July 2025) to identify relevant literature from 2016 to 2025. Studies that describe EPAs implemented in pharmacy programs, assess the perspectives on EPAs implemented, or evaluate student performance on EPAs were included. Studies that did not assess actual experience of EPAs’ implementation were excluded. The data were described narratively and through frequencies and tables. Twenty-four studies were included in the review. Most studies pointed out that the EPAs’ framework has been adopted mostly in practice experiences, but with a few experiences in course activities. The most frequent approach to assess student performance on EPAs was direct practice observation. Student performance on EPAs improved after participating in educational activities. From the perspectives of students, preceptors, and faculty members, EPAs facilitated student assessment of competencies; however, there are challenges in their implementation, such as heavy workload. The findings of this review can inform faculty members and health professionals who intend to implement EPAs’ framework.

## 1. Introduction

Competency-based education (CBE) has become a common approach in health professions education, including pharmacy education. This model centers on the ongoing development of students’ knowledge, skills, and attitudes throughout their academic programs [1]. However, assessing learners within CBE frameworks can sometimes be challenging due to the abstract nature of competency measurement. Despite its advantages, evaluating learners within CBE can be complicated, especially because competencies are often abstract and difficult to measure directly. To address these complexities, Entrustable Professional Activities (EPAs) were introduced to bridge the gap between clinical practice demands and competency assessment [2]. EPAs define specific tasks or responsibilities that students are authorized to perform after they have demonstrated appropriate competency [3,4]. The EPA model aimed at making competency expectations clearer and more relevant, outlining specific tasks students should be able to perform and gradually preparing them for independent clinical responsibilities [2,4]. Students are granted more autonomy as they show readiness to handle more complex responsibilities, with a structured entrustment process that evolves with their development of competencies [2]. EPA entrustment is typically described in five levels. At the first level, students only observe and do not participate directly. The second level allows students to perform tasks under immediate and direct supervision. At the third level, students carry out duties with supervision available when needed, rather than continuously present. Level four allows for independent performance with only distant supervision. At the highest, fifth level, students are trusted to supervise less experienced colleagues [5].

After its introduction in 2005, the EPA framework was initially used in medical residency training and later adapted for undergraduate medical education [3]. Its adoption has since spread to other health professions such as pharmacy, aiming to align assessment methods more closely with practical, real-world demands [6]. In the United States of America (USA), the American Association of Colleges of Pharmacy (AACP) established a set of Core Entrustable Professional Activities for New Pharmacy Graduates (Core EPAs) in 2016 to operationalize educational outcomes previously established by Center for the Advancement of Pharmacy Education (CAPE) [7]. These Core EPAs were updated in 2022, reducing the number from 15 to 13 [8]. Within this framework, there are five supervision levels, and pharmacy students are expected to achieve at least the third level—reactive supervision—by the time they graduate [7]. EPAs have also been developed for postgraduate pharmacy training, including residency programs in the USA [9] and specialization in the Netherlands [10]. In Australia, the development and validation of EPAs for pharmacy intern training have been documented, with the Australian Pharmacy Council recognizing EPAs as a recommended workplace-based assessment method for interns [11]. The EPA framework has also been applied to professional development for practicing pharmacists and pharmacy staff, such as the creation of an EPA focused on medication history-taking for workplace training in Australian hospitals [12].

Research on EPAs in pharmacy education has primarily addressed the creation and validation of EPA statements [11,13]. However, there are relatively few studies that detail how EPAs are put into practice or evaluated within pharmacy programs [11,13]. Some authors suggest that future research should place greater emphasis on the implementation and evaluation of EPAs, ensuring they are used effectively to enhance student assessment and competency development [11,13]. A literature review found only limited examples of EPAs being applied in practice and did not offer insights into their integration in teaching, assessment, or student performance outcomes [13]. Summarizing these gaps could provide valuable guidance to educators and health professionals seeking to adopt the EPA framework in support of competency-based education.

This scoping review describes how Entrustable Professional Activities (EPAs) have been implemented in pharmacy education, including how EPAs are utilized in teaching and assessment, factors that promote or hinder their use, recommendations for implementation, and evidence of student performance where EPAs have been put into practice.

## 2. Methods

This scoping review was developed based on the Joanna Briggs Institute (JBI) methodology for scoping reviews [14] and in line with the Preferred Reporting Items for Systematic Reviews and Meta-Analyses extension for Scoping Reviews (PRISMA-ScR) [15]. This scoping review protocol was registered 24 June 2025, on Open Science Framework registries and can be accessed here https://osf.io/2xat5 (accessed on 15 September 2025). This review was performed in accordance with the PRISMA (Preferred Reporting Items for Systematic Reviews and Meta-Analyses) guidelines. Microsoft 365 Copilot was utilized during the preparation of this manuscript to enhance the clarity, coherence, and overall quality of the writing and language used throughout the document.

### 2.1. Search Strategy

The search strategy aimed to identify published studies from peer-reviewed journals. As recommended by JBI methodology, an initial limited search of MEDLINE (PubMed) was conducted to identify relevant articles and guide the final search approach, which was then adapted for Scopus. Unlike what was outlined in the scoping review protocol, the Web of Science database was not included, as the researchers lost access to it during the search process. Searches in MEDLINE (PubMed) and Scopus were performed on 08 July 2025. To broaden coverage, gray literature from Google Scholar was included. The search terms covered various denominations for pharmacy students or students, Entrustable Professional Activities (EPA), and implementation-related topics. Table 1 presents the search strategy.

### 2.2. Inclusion and Exclusion Criteria

A PCC (Population/Concept/Context) framework was developed to inform the inclusion and exclusion criteria, considering pharmacy programs as population, EPAs as the concept, and implementation as the context. The inclusion criteria were studies that describe EPAs implemented in pharmacy programs or assess the perspectives of different stakeholders (preceptors, faculty, and students) on EPAs implemented or evaluate student performance on EPAs. The papers that met at least one of these inclusion criteria were included in the review. Table 2 presents the inclusion and exclusion criteria.

Pharmacy education included educational programs leading to registration as a pharmacist based on universities or colleges. Examples of programs in pharmacy education included Doctor of Pharmacy (PharmD), Master of Pharmacy (MPharm), and Bachelor of Pharmacy. The scope of this review did not cover professional development initiatives for pharmacists and pharmacy staff and postgraduate-level experiences such as residency or specialization programs. Additionally, pre-registration internships and training courses for provisionally registered pharmacists—frequently found in countries like Australia and the United Kingdom—were excluded. Experiences of implementation in different contexts were included, such as courses, practice experiences (e.g., Introductory Pharmacy Practice Experience-IPPE and Advanced Pharmacy Practice Experience-APPE), and throughout the curriculum of pharmacy programs. Studies that did not assess an actual experience of EPA implementation were not included. Studies were not included if they focused only on creating and validating EPAs without implementing them, or if they assessed EPA quality without addressing how they were used in practice or considering the perspectives of key stakeholders. Guidelines for the elaboration and implementation of EPAs were also excluded from the literature reviews.

This review included studies that assessed the perspectives of different stakeholders (preceptors, faculty, and students) on EPAs implemented. Examples of such studies included assessment of stakeholders’ perceptions of facilitators and barriers in using EPAs, and recommendations for the use of EPAs. Perceptions of EPAs not actually implemented were not considered in this review.

Evaluations of student performance on EPAs implemented were included in the review. Student assessment could be presented through entrustment levels (one to five) or other measures of student performance on EPAs. Non-EPA based evaluations of student learning were excluded from this review.

Studies were limited to English, Portuguese, and Spanish, reflecting the authors’ language capabilities, and to publications from 2016 onward due to the creation of the Core Entrustable Professional Activities for New Pharmacy Graduates (Core EPAs) of the USA in 2016 [7]. The review considered diverse types of studies, such as experience reports, program descriptions or evaluations, observational studies, experimental or quasi-experimental studies, and qualitative and mixed-methods studies.

### 2.3. Data Selection

After searching MEDLINE (PubMed) and Scopus, all identified records were collated and uploaded into EndNote v.X9, and duplicates were removed. Collated titles and abstracts searched in MEDLINE (PubMed), Scopus, and Google Scholar were compiled in an inclusion/exclusion MS Excel spreadsheet, with exclusion of duplicates. Following a pilot test, titles and abstracts were screened by two independent reviewers (LCOAS and LFAR) for assessment against the inclusion criteria for the review. The Kappa coefficient was calculated to measure agreement between the two reviewers. Disagreements between the reviewers were resolved with a third reviewer (MGL). The results of the search were presented in a PRISMA flow diagram [15].

### 2.4. Data Extraction

Data were extracted from papers included in the scoping review using a data extraction tool developed by one of the reviewers (MGL) to collect data. Data extracted from the publications that met the inclusion criteria were: authors, country, year of publication, study design, study aims, characteristics of EPAs, type of educational activity and level of education in which EPAs were used, use of EPAs in teaching activities and assessment, facilitators and barriers of use of EPAs, suggestions to improve the use of EPAs, student performance in carrying out the EPAs, and main findings of the study. The three reviewers (LCOAS, LFAR, and MGL) extracted the data and discussed the findings, and any disagreements were resolved through consensus. Data analysis was performed using Microsoft 365 (Excel for Office 365). The data were described narratively and through frequencies and tables.

## 3. Results

**Figure 1:**
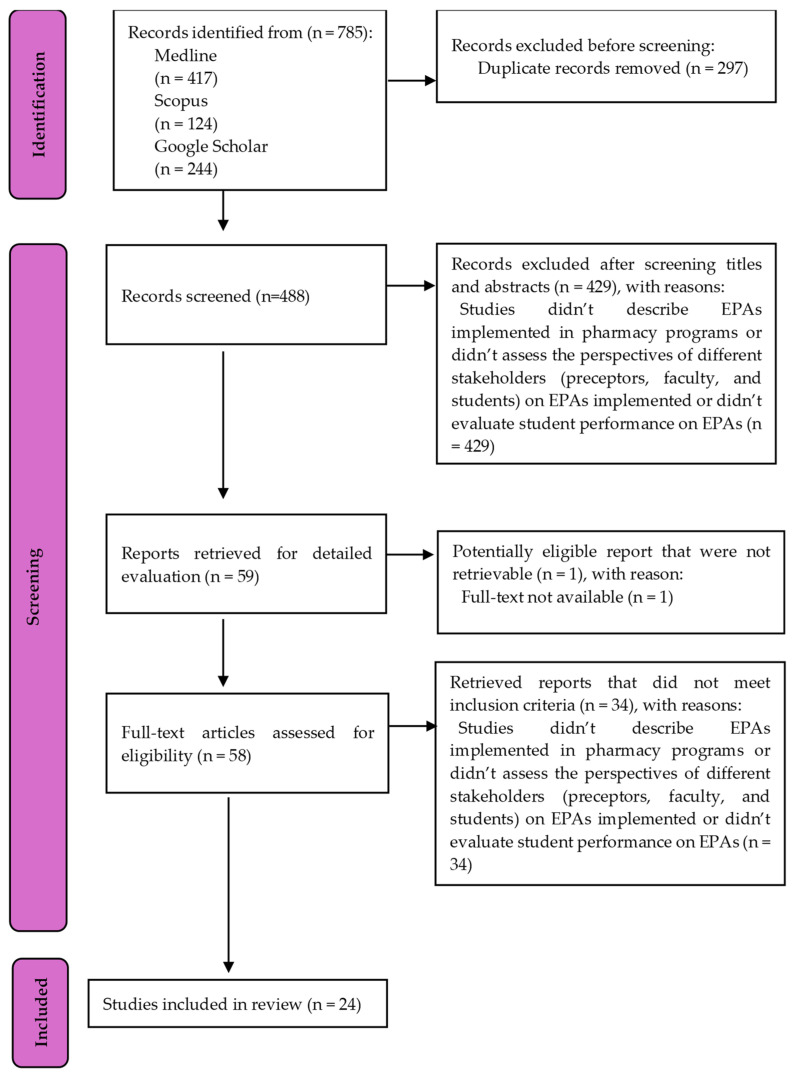
PRISMA flow diagram for study selection.

A total of 24 studies were selected for inclusion in this review from an initial pool of 785 articles identified through database searches and gray literature, as illustrated by the PRISMA-ScR flow diagram (Figure 1). The agreement between the two reviewers during study selection was high, with a Kappa coefficient of 0.93. Key features of the selected studies are summarized in Table S1.

Table 3 reveals that the majority of these studies (87.5%) were conducted in the USA. Over half of the studies (54.2%) were published between 2022 and 2025.

### 3.1. Use of EPAs in Teaching and Learning

The studies reviewed implemented Entrustable Professional Activities (EPAs) in various pharmacy education programs: PharmD (21 studies in the USA and one in Lebanon), Bachelor of Pharmacy (one study in Australia), and Master of Pharmacy (one study in the United Kingdom). According to Table 4, most studies (n = 16) in the USA assessed the core EPAs endorsed by the AACP for new pharmacy graduates established in 2016 [16,17,18,19,20,21,22,23,24,25,26,27,28,29,30]. In ten studies, only a subset of the 15 core EPAs was addressed; in nine of these, the activity “Create a written plan for continuous professional development” was omitted. Six studies described EPAs tailored specifically for educational programs, such as those developed for placements that included patient counseling [31] and a list of EPAs developed for pharmacy practice experiences of pharmacy programs in the USA before the publication of the core EPAs [32,33,34,35,36]. Three studies described a particular EPA or supporting task, for example, medicine dispensing [37] or applying evidence-based medicine [38].

Educational activities involving EPAs included practice experiences [17,18,19,21,23,25,26,27,30,31,32,33,34,35,36] and courses with simulations [20,22,24,28,37,38,39]. The practice experiences were IPPEs and APPEs in PharmD programs from the USA, and one placement of Master of Pharmacy (Mpharm) in the United Kingdom [31].

Literature described the development of EPA-based assessment tools, training for faculty, preceptors, and students, and pilot testing with student samples or subsets of EPAs [17,18,19,33]. According to two studies in the USA, rubrics for preceptors to evaluate student performance on EPAs during practice experiences demonstrated high internal consistency [18,33].

Various methods were used to assess EPA performance, with direct practice observation by preceptors during practice experiences being the most common (79.2%). Other approaches included structured self-assessment by students (12.5%), product evaluation (12.5%), case-based discussion or case-based study (8.3%), and reflective activity (8.3%). Five studies reported using more than one EPA-based method for teaching and assessment.

### 3.2. Facilitators, Barriers, and Recommendations to Improve the Use of EPAs

Ten studies assessed facilitators or barriers in using EPAs from the perspective of students, preceptors, and faculty members, and these findings are presented in Table 5. The most frequent facilitator was facilitating student assessment of competencies (n = 5). For example, preceptors noted that these tools help set clear goals and identify both strengths and areas for student improvement [26,27]. One study indicated that involving preceptors in the design of EPA-related tasks and assessment rubrics improved their acceptance of these tools [27].

The most reported barriers were difficulties in using EPA-based instruments for assessment (n = 7) and heavy workload (n = 3). The concept of “entrustability” was found to be abstract and challenging for both preceptors and students to grasp [26]. There was also noted variation among preceptors in how they assessed student performance on EPAs. One study analyzed feedback provided by preceptors on EPAs performed by students during IPPEs and found both synthetic and detailed comments about students’ development [34]. Some preceptors commented that certain EPAs were not relevant to their particular work settings, making assessment difficult. In one study, 7.6% of preceptors disagreed that EPAs from the patient care provider domain were appropriate for completion by students during a community pharmacy IPPE [27]. Faculty members, preceptors, and students informed that EPA-based assessments are time-consuming, generating heavy workload. Recommendations to address these challenges included adopting more user-friendly EPA assessment tools and providing additional training on EPAs and their assessment.

### 3.3. Student Performance on EPAs

Student performance on EPAs was evaluated in 20 (83.3%) of the studies, generally showing improvement following educational interventions such as courses and practical experiences.

In the context of practice experiences, 11 studies (45.8%) reported EPA scores or entrustment levels. Mean student EPA scores of three or higher were found following APPEs, and growth in clinical EPA scores related to direct patient care [18,19,21,23,32].

Two studies tracked student performance on EPAs throughout the duration of PharmD programs [16,29]. As students advanced in their studies, their confidence in performing EPA tasks increased, and they needed less supervision [16,29]. One study specifically noted that entrustment and supervision levels improved across all EPAs as students participated in a combination of skill-based lab courses and practice experiences in various settings [29].

## 4. Discussion

This review compiles current knowledge regarding the integration of Entrustable Professional Activities (EPAs) within pharmacy education, offering insights into how EPAs are utilized for teaching, assessment, and gauging student performance. It also examines factors that help or hinder EPA adoption and provides recommendations for improving the implementation process.

Most studies included in this review focused on the description or evaluation of EPAs in pharmacy programs across the USA. Since 2016, the American Association of Colleges of Pharmacy (AACP) has advocated for the inclusion of core EPAs in PharmD curricula and has provided a guide outlining recommended steps for implementation [40]. Some implementation steps suggested by this guide were reported by included studies in this review, such as establishment of a faculty team to lead the implementation, adopting existing curricular assessments for EPAs’ milestones, orientation for faculty members, preceptors, and students in using of EPA-based assessment tools, and piloting the EPAs’ assessments with students [17,18,19,33]. Notably, some programs chose not to assess the core EPA “EPA15-Create a written plan for continuous professional development”. According to a study about quality assessment of core EPAs, this EPA about professional development did not meet the minimum cut-off scores in domains that define one EPA: discrete unit of work, entrustable and essential task of one profession, and curricular role [41]. In the update of core EPAs that occurred in 2022, the core EPA 15 was excluded [8]. Planning and monitoring their own professional development are important tasks to be performed by pharmacy students, but they may be better assessed through non-EPA based methods, such as learning portfolios.

One study suggests that despite true assessment of entrustability can only occur in workplace-based situations, the pharmacy educators have designed EPA-based simulated activities as a bridge to practice experiences [24]. Programs that combined a sequence of simulated EPA courses with hands-on practice saw increases in students’ readiness to be entrusted with professional responsibilities [29].

In this review, direct observation of students in practice was the predominant EPA-based assessment method. Other approaches included case-based discussions and evaluation of written products. Using a mix of assessment techniques—beyond just direct observation—was found to provide a more comprehensive view of student capabilities and reduce evaluator bias [3]. Assessment methods were typically tailored to fit the specific EPA being evaluated. For example, case-based discussions were adopted to evaluate pharmacotherapy-related cases [31] and product evaluation to assess written materials filled during medication review [21].

Faculty members, preceptors, and students generally felt that EPAs clarified expectations and standardized the assessment of student performance, fulfilling a major goal of the EPA framework [3]. Involving preceptors in the design of EPA-based evaluation tools increased acceptance and facilitated smoother implementation, a trend observed both in the USA and in a study about pharmacy intern training programs in Australia [5].

Different stakeholders on EPAs’ implementation informed barriers in using EPAs and suggestions to improve this framework. One recurring issue was the subjectivity involved in using EPA-based assessment instruments; some preceptors may have assessed students based on personal standards rather than the criteria specified in EPA tools [26,33]. This highlights the need for ongoing training and soliciting feedback from preceptors. According to one study, an assessment tool was redesigned in response to preceptor input: the new format employed EPA entrustment levels for formative feedback and standardized written narratives—including illustrative vignettes—for summative evaluation [36].

Some preceptors, particularly in community pharmacy settings, noted that certain EPAs did not align with their daily practice [27]. For example, patient care-related EPAs were sometimes not applicable in these environments, with a survey indicating that in 16% of community pharmacy practice experiences, students did not engage in patient care activities [42]. According to a review about community pharmacy practice experiences, the most common activity was dispensing and tasks related to implementing and follow-up a care plan were less frequent [43]. One suggestion to address these problems is to adapt EPAs to better fit the context of specific practice settings. For example, in one pharmacy program described in this review, core EPAs related to implementing and follow-up a care plan are not mandatory in community pharmacy practice experiences, while hospital practice experiences did not include identifying patients at risk for prevalent diseases and ensuring immunizations of patients [29].

Student performance tended to improve after participating in EPA-based educational activities. In the practice experiences of the USA, students showed EPA scores equal to or higher than 3 (reactive supervision), which is required by the AACP [7]. These high scores could reflect genuine increase in student performance after EPAs’ implementation or difficulties of preceptors in using the EPAs’ tools; further research is needed to clarify these possibilities. In this review, some studies observed enhanced student competencies in clinical EPAs after completing practice experiences, with pharmacy programs increasingly offering opportunities for interprofessional education and simulated patient interactions [19,29].

This review has some limitations. Some characteristics of the educational activities and methods of assessment may have been considered absent when, in fact, they were not described in the studies. Only studies published in English were included, although the authors considered in the search strategy the Spanish and Portuguese languages. As a result, some relevant literature may not have been included.

## 5. Conclusions

This review showed that EPAs have been implemented in pharmacy education to operationalize competency-based education, especially in pharmacy programs in the USA. Most studies described the implementation of EPA-based assessments in practice experiences, with the direct practice observation the most common method for assessment. Implementation experiences highlighted both facilitators and barriers to EPA adoption. Stakeholders agreed that EPAs aided in competency-based student evaluation, though heavy workloads and other challenges persist. Student performance improved after participating in EPA-based educational activities. For faculty members and health professionals considering EPAs’ implementation, this review recommends ongoing preceptor and student training, utilizing holistic assessments informed by user feedback, and monitoring student progress across the curriculum.

## 6. Future Directions

This review expands the scientific literature on the status of EPA implementation in pharmacy education. Future research should address existing gaps, such as the use of EPAs in postgraduate pharmacy programs and professional development for pharmacists and staff. Expanding studies beyond the context of the USA and conducting longitudinal research could provide deeper insights into how educational design and assessment methods influence student performance. Exploring the perspectives of patients and healthcare professionals could further illuminate whether EPAs achieve their goal of minimizing risks in practice.

## Figures and Tables

**Table 1:** Summary of research protocols.

Database	Protocol	Notes	Date
Medline (Pubmed)	((((((((((Education, Pharmacy[Title/Abstract]) OR (Students, Pharmacy[Title/Abstract])) OR (Pharmaceutic*[Title/Abstract])) OR (Pharmaceutical Education[Title/Abstract])) OR (Pharmacy Education[Title/Abstract])) OR (Pharmacy Student[Title/Abstract])) OR (Pharmacy Students[Title/Abstract])) OR (pharmacy[Title/Abstract])) OR (pharmacist[Title/Abstract])) AND ((((((((Implementation[Title/Abstract]) OR (implement*[Title/Abstract])) OR (Evaluation[Title/Abstract])) OR (outcome*[Title/Abstract])) OR (assessment[Title/Abstract])) OR (performance[Title/Abstract])) OR (perspective*[Title/Abstract])) OR (perception*[Title/Abstract]))) AND (((entrustable professional activities[Title/Abstract]) OR (EPA[Title/Abstract])) OR ((entrustable professional activity[Title/Abstract]))	Time-restricted research (2016 to 2025); language (English, Portuguese and Spanish	8 July 2025
Scopus	“Education, Pharmacy” OR “Students, Pharmacy” OR “Pharmaceutic” OR “Pharmaceutical Education” OR “Pharmacy Education” OR “Pharmacy Student” OR “Pharmacy Students” OR “pharmacy” OR “pharmacist” AND “Implementation” OR “implement” OR “Evaluation” OR “outcome” OR “assessment” OR “performance” OR “perspective” OR “perception” AND “entrustable professional activitie” OR “EPA” OR “entrustable professional activity”	Time-restricted research (2016 to 2025); language (English, Portuguese and Spanish)	8 July 2025
Google Scholar	Education OR Pharmacy OR Pharmacist “entrustable professional activities” OR “entrustable professional activity”	Search ordered by relevance; time-restricted research (2016 to 2025)	8 July 2025

The symbol * was used for truncation in the search.

**Table 2:** Inclusion and exclusion criteria in the review.

Inclusion criteria	Studies that describe EPAs implemented in pharmacy education or assess the perspectives of different stakeholders (preceptors, faculty, and students) on EPAs implemented or evaluate student performance on EPAs Studies in English, Portuguese, and Spanish languages Diverse types of studies, such as experience reports, program descriptions or evaluations, observational studies, experimental or quasi-experimental studies, and qualitative and mixed-methods studies Studies published from 2016 to 2025
Exclusion criteria	Studies about professional development initiatives for pharmacists and pharmacy staff, postgraduate-level experiences, and pre-registration internships for provisionally registered pharmacists Studies that did not describe or assess an actual experience of EPA implementation Studies that assessed perceptions of EPAs not actually implemented Studies that assessed student learning but did not adopt EPAs in evaluations

**Table 3:** General characteristics of the studies included in the review (*n* = 24).

Characteristics	Number of Papers (%)
Year of publication	
2019–2021	11 (45.8)
2022–2025	13 (54.2)
Country	
USA	21 (87.5)
Australia	1 (4.2)
Lebanon	1 (4.2)
United Kingdom	1 (4.2)
Study design	
Quantitative	17 (70.8)
Mixed-methods	4 (16.7)
Qualitative	2 (8.3)
Experience report	1 (4.2)

**Table 4:** Use of EPAs in teaching and student assessment.

Variables	Number of Papers (%)
EPAs assessed	
Core EPAs recommended by the AACP for new pharmacy graduates in the USA	16 (66.7)
List of EPAs designed specifically for educational programs	6 (25.0)
One specific EPA or supporting task	2 (8.3)
Type of educational activity with EPAs implemented	
Practice experiences	15 (62.5)
Courses	7 (29.2)
A sequence of skill lab-based courses and practice experiences	1 (4.2)
All the four professional years from one PharmD program	1 (4.2)
Use of EPAs in teaching and assessment	
Direct practice observation in practice experiences by preceptors	19 (79.2)
Student self-assessment of performance through a structured form	3 (12.5)
Product evaluation	3 (12.5)
Case-based discussion or case-based study	2 (8.3)
Reflective activity	2 (8.3)
Not informed	1 (4.2)

**Table 5:** Facilitators, barriers, and recommendations to improve the use of EPAs.

Variables	Number of Papers (%)
Facilitators	
Facilitate student assessment of competencies	5 (20.8)
Facilitate assigning grades to the students	3 (12.5)
Facilitate feedback to the students	1 (4.2)
Provide additional opportunities for preceptor development	1 (4.2)
Barriers	
Difficulties in using EPA-based instruments for assessment	7 (29.2)
Heavy workload	3 (12.5)
Some EPAs are not applicable to workplace settings	3 (12.5)
Insufficient faculty involvement and resource constraints	1 (4.2)
Recommendations	
Adopt more user-friendly EPA-based assessment tools	3 (12.5)
Decrease the number of assessments	1 (4.2)
Additional training in the EPAs’ framework and EPA-based assessments	1 (4.2)

## Data Availability

No new data were created or analyzed in this study. Data sharing is not applicable to this article.

## Supplementary Materials

The following supporting information can be downloaded at: https://www.mdpi.com/article/10.3390/pharmacy13060156/s1, Table S1: Data from studies included in the review.
