# Supplementary material for: How Have Entrustable Professional Activities (EPAs) Been Implemented in Pharmacy Education? A Scoping Review
Source: Pharmacy (Basel). 2025 Nov 1;13(6):156. doi: 10.3390/pharmacy13060156 (PMC12641839; doi:10.3390/pharmacy13060156)
Supplement: Supplementary file 1 [file pharmacy-13-00156-s001.zip › pharmacy-3915761-supplementary.pdf]

Table S1. Data from studies included in the review.

Authors; Year; Country	Main objectives	Type of educational activity	Characteristics of EPAs	Use of EPAs in teaching and assessment of student performance	Main findings of the study
Rhodes *et al.*, 2019 [1]; United States of America (USA)	To examine entrustable professional activities (EPAs) as an assessment tool for student pharmacists completing early practice experiences	Early practice experiences in a community pharmacy or health system pharmacy setting, entitled "Immersion Experience 1"	Fourteen EPAs developed for early pharmacy practice experiences of the pharmacy program at the University of North Carolina Eshelman School of Pharmacy	Preceptors observe student performance, provide feedback, and fill in a structured form at the end of the first and second month of the practice experience. Students assessed their own performance and fill structured form. For a student pharmacist to successfully pass the course, a level of “marginal” (Level 2.0) or greater must be achieved for EPA1 (review and collect pertinent medication and medical information), EPA2 (perform a comprehensive medication history interview), EPA8 (document clinical encounters), EPA11 (provide an oral presentation of a clinical encounter to a pharmacist or health care provider), and EPA12 (form clinical questions and retrieve evidence to advance patient care). All practice sites were required to participate in a live webinar hosted by the course directors prior to the launch of the experience. The webinar described coursework students had completed to date, expectations, and oriented preceptors to the appropriate use of the UNC EPA Statements and Clinical Evaluation Scale.	There was an increase in student performance over time. The EPA statements may be a reliable assessment tool for student performance in pharmacy education.

Table S1. Data from studies included in the review (continued)

Authors; Year; Country	Main objectives	Type of educational activity	Characteristics of EPAs	Use of EPAs in teaching and assessment of student performance	Main findings of the study
Pittenger *et al.*, 2019 [2]; United States of America (USA)	To determine pharmacy students' perceptions and self-reported preparedness to perform the 15 core entrustable professional activities (EPA) established by the American Association of Colleges of Pharmacy for new pharmacy graduates	All the four professional years from the six-year PharmD program	All the 15 US Core Entrustable Professional Activities for new pharmacy graduates 2016	Not informed	The core EPA statements were consistently rated by pharmacy students as relevant to pharmacy practice and as an expectation in multiple settings. Students perceived that they require less supervision when performing EPAs as they progressed through the curriculum.
Lounsbery, et al., 2019 [3]; United States of America (USA)	To determine if the number of patient encounters during advanced pharmacy practice experiences (APPEs) relates to student self-assessment of patient care skills using entrustable professional activities (EPAs)	Practice experiences (APPEs) in clinical settings (acute care/institutional, community pharmacy and ambulatory care)	A list of EPAs developed for practice experiences of the pharmacy program at the University of Minnesota College of Pharmacy, Minneapolis, Minnesota	Preceptors observe student performance, provide feedback, and fill a structured form during midpoint and final APPE evaluations.	Tracking student patient encounters provided insight into the quantity and variety of patients and conditions seen and level of care provided by students during APPEs. Mean scores on EPAs increased over time with increased exposure to patients. Patient tracking can be used to inform the curriculum by identifying potential gaps in both didactic and experiential education.

Table S1. Data from studies included in the review (continued).

Authors; Year; Country	Main objectives	Type of educational activity	Characteristics of EPAs	Use of EPAs in teaching and assessment of student performance	Main findings of the study
Rivkin *et al.*, 2020 [4]; United States of America (USA)	To describe the design and evaluation of a program implemented to ready clinical faculty members to use entrustable professional activities (EPAs) for teaching and assessment in experiential education	Practice experiences (APPEs) in ambulatory care and acute care	Three EPAs from US Core Entrustable Professional Activities for new pharmacy graduates 2016 were chosen for the pilot program: collect, assess, and information master	The group recommended that the faculty development program held prior to the pilot implementation of EPAs in the curriculum occur over two sessions. The first session (1.5 hours) offered a general overview of the EPA framework. The second session (4 hours) was case based. Faculty members assigned ratings at midpoint and at the end of each APPE block for each student precepted using the entrustment rubric; students rated level 3 or higher by the end of an APPE block were deemed entrusted. The EPA ratings were not used in the current APPE grading structure.	The faculty development program was effective in preparing faculty members to use an entrustment rubric and teach and evaluate students during the pilot implementation of EPAs. The authors plan to deliver similar programming to preceptors, keeping successful elements while addressing the lessons learned from delivering the program to clinical faculty members.
Smith *et al.*, 2020 [5]; United States of America (USA)	This paper describes the development and pilot implementation of the EPA assessment within the curriculum's core advanced pharmacy practice experiences (APPEs)	Practice experiences (APPEs) in general medicine, ambulatory care, institutional, and community settings	14 EPAs from the Core Entrustable Professional Activities for new pharmacy graduates in the USA (2016 version) with four additional program-specific EPAs developed by the college	Preceptors observe student performance, provide feedback, and fill a structured form. Each EPA had six options from which the preceptor could select: five levels of entrustment and a not applicable (N/A) option. Those who met or exceeded the expected performance level for a given EPA were awarded 100% of points for that EPA. Students who did not meet the expected level for an EPA were awarded a proportional percentage of points for that EPA.	The EPA assessment tool is a reliable and valid instrument for assessing EPA achievement in the APPE year. Future work should focus on determining the longitudinal utility of the EPA tool by comparing outcomes in introductory and advanced pharmacy practice experiences.

Table S1. Data from studies included in the review (continued).

Authors; Year; Country	Main objectives	Type of educational activity	Characteristics of EPAs	Use of EPAs in teaching and assessment of student performance	Main findings of the study
Croft *et al.*, 2020 [6]; Australia	Describe the inclusion of an EPA scale in a simulated medicine dispensing activity for evaluating readiness of final year undergraduate students for supervised practice following graduation	A course (Transition to Practice) conducted across the last two semesters in the fourth year of the University of Newcastle Bachelor of Pharmacy (BPharm[Hons]) program	One EPA for medicine dispensing developed by the authors	The simulations were designed to represent a patient encounter in supply of a prescribed medicine, and the performance of students was evaluated by assessors (faculty members) using an entrustment scale, with debriefing and feedback process. All assessors received training prior to participation in the study. Assessors were initially provided with a package outlining the research project, the theoretical basis for the assessment methodology, an outline of the simulation scenarios, and a detailed description of the assessment framework containing an entrustment scale. Assessors then had a one-to-one briefing either in person or via teleconference that discussed how to use the assessment tool.	The findings revealed to educators that entrustment decisions using trained experts are a suitable way of expressing students' ability to perform clinical tasks. EPAs can provide clarity and flexibility for learners and enhance the utility of assessment methods for evaluating competence in medicine supply.

Table S1. Data from studies included in the review (continued).

Authors; Year; Country	Main objectives	Type of educational activity	Characteristics of EPAs	Use of EPAs in teaching and assessment of student performance	Main findings of the study
Marshall *et al.*, 2020 [7]; United States of America (USA)	To evaluate levels of entrustability and practice readiness in advanced pharmacy practice experience (APPE) students using a pilot instrument designed to assess their competency in performing the entrustable professional activities (EPAs) expected of new pharmacy graduates	Practice experiences (APPEs) in clinical settings	Fourteen EPAs from the Core Entrustable Professional Activities for new pharmacy graduates in the USA (2016 version)	Preceptors observe student performance, provide feedback, and fill a structured form at APPE midpoint and end. There is self-assessment of student performance through a structured form at APPE midpoint and end. The pilot instrument assigned equal weights to each EPA and levels one to five so that the total score would be 70 if all 14 items were ranked level five (ie, 5 x 14=70). Total scores were normalized to 100, with adjustments made if a response of not applicable was given.	Pharmacy students' proficiency in EPA improved during individual APPEs. According to preceptors, students' greatest improvement in entrustability was in educating patients and colleagues regarding appropriate use of medications and collecting information to identify medication-related problems.
Law et al., 2021 [8]; United States of America (USA)	To design and assess the outcomes of a longitudinal, integrated pharmacy course	A one-credit course, Applications of Pharmacy Practice, meant to integrate all the semester's coursework into extended cases and team activities	Core Entrustable Professional Activities for new pharmacy graduates in the USA (2016 version)	EPA-based rubric was developed for each course session's team activity. Students in their first professional year of the PharmD program were expected to perform at level 1 (observation). Each session concluded with a one-hour debrief, which allowed time to discuss the solutions to the individual case and the team activity.	The addition of a longitudinal integrated course, Applications of Pharmacy Practice, appeared to benefit students, helping them integrate material from all their courses during the semester. This integration of clinical, administrative, and pharmaceutical sciences material reduced compartmentalization of knowledge.

Table S1. Data from studies included in the review (continued).

Authors; Year; Country	Main objectives	Type of educational activity	Characteristics of EPAs	Use of EPAs in teaching and assessment of student performance	Main findings of the study
Borja-Hart *et al.*, 2021 [9]; United States of America (USA)	The objective of this study was to describe an EPA supporting task aimed at improving utilization of evidence-based medicine skills and its impact on student self-perceived confidence, rating of skills needed, and competence	An activity (The mock Pharmacy and Therapeutics committee) integrated into the third-year course Interprofessional Education and Clinical Simulation V	One EPA supporting task aimed at improving students' utility of evidence-based medicine	There is a product evaluation. A grading rubric adapted from Law et al.6 scored student groups' monographs and presentations. The maximum points for the monograph and presentation combined was 30 (24 for the monograph and six for the presentation). The course director (NBH) evaluated all submissions. The activity accounted for 5% of the final grade in 2017 and 9% in 2018. Groups voted for the drug to be added to formulary using an online, game-based learning platform. Self and peer assessment was conducted at the end of the activity.	A mock pharmacy and therapeutics committee activity represents an innovative way to evaluate an EPA supporting task that allows students to improve their evidence-based analysis, written, and verbal communication skills. Students also exhibited improved confidence in their writing skills and drug monograph preparation. These skills can transcend into their experiential experience and into future practice.
Sjoquist *et al.*, 2021 [10]; United States of America (USA)	To explore and evaluate open-ended feedback on entrustable professional activities (EPAs) provided by preceptors to Doctor of Pharmacy (PharmD) students completing their first practice experience	Early practice experiences in a community pharmacy or health system pharmacy setting	Fourteen EPAs developed for early pharmacy practice experiences of the pharmacy program at the University of North Carolina Eshelman School of Pharmacy	Preceptors observe student performance, provide feedback, and fill in a structured form at the end of the first and second month of the practice experience. Students assessed their own performance and fill structured form.	This study provides valuable insight into optimizing preceptor-provided written feedback on EPAs. Conducting deeper analysis of preceptor feedback using focus groups or structured interviews is suggested to further explore preceptors' provision of EPA assessment to student pharmacists practicing in real-world settings.

Table S1. Data from studies included in the review (continued).

Authors; Year; Country	Main objectives	Type of educational activity	Characteristics of EPAs	Use of EPAs in teaching and assessment of student performance	Main findings of the study
Rathbone *et al.*, 2021 [11]; United Kingdom	To explore the quality and utility of undergraduate pharmacy students using entrustable professional activities to provide patient counselling services in secondary care	Practice experience (placement) of Master of Pharmacy (Mpharm) in hospital setting	Three EPAs designed for the placement that included patient counseling	The intervention in placement focused on students visiting a single hospital for 3 h, each week, for ten weeks to complete medication histories and provide medication counselling using EPAs that covered three therapeutic areas i) inhaler technique, ii) simple analgesia and iii) non-vitamin K anticoagulants. During placements, a model of direct supervision was used which included peer supervision (student to student), remote supervision (accessing a registered clinical pharmacist via telephone) and direct clinical supervision (by a placement facilitator observing activity). Records of each consultation completed by students were made via a proforma that used a subjective, objective, assessment and plan (SOAP note) structure. Product evaluation and case-based discussions were carried out to assess performance on EPAs, after each of the 10 sessions of the placement.	The findings indicate that EPAs are deployable within a pharmacy setting and that students were able to contribute to the pharmaceutical care of patients and refer patients on for follow-up by a clinical pharmacist when necessary.
Eudaley *et al.*, 2022 [12]; USA	To describe a unique student-driven transitions-of-care (TOC) documentation process within an inpatient family medicine advanced pharmacy practice experience (APPE) and to evaluate student-perceived growth in autonomy in selected EPAs and supporting tasks	Practice experience (APPE) in inpatient family medicine	Five EPAs from the Core Entrustable Professional Activities for new pharmacy graduates in the USA (2016 version)	Product evaluation. Student pharmacists participated in interdisciplinary bedside rounds on acutely ill adult inpatients receiving care from the inpatient family medicine teaching teams. Notes were generated for patients discharged from the inpatient service. After the student completed the note, the preceptor (pharmacy resident or faculty) reviewed the note in the clinic for the hospital follow-up appointment.	The importance of pharmacist and pharmacy trainee involvement in the TOC process has been well-documented. Involving students in student-driven TOC documentation processes serves to facilitate student-perceived growth in autonomy within selected EPAs.

Table S1. Data from studies included in the review (continued)

Authors; Year; Country	Main objectives	Type of educational activity	Characteristics of EPAs	Use of EPAs in teaching and assessment of student performance	Main findings of the study
Schroeder *et al.*, 2023 [13]; United States of America (USA)	This study examined EPAs as a feedback tool for student pharmacists in a skills lab series	A six-semester pharmacy skills development laboratory course, including high-stakes assessments, such as blood pressure or practical calculations, and OSCE stations	Thirteen EPAs from the Core Entrustable Professional Activities for new pharmacy graduates in the USA (2016 version)	An individual report showing the level of entrustment generated based on performance (minimum competency met/not met) of high-stakes activities and OSCEs is provided to students each semester by faculty members. The framework was established with the intent to track student progress and to provide feedback to students throughout the skills lab series.	This study demonstrates the potential to use EPAs as a feedback tool for student pharmacists in a skills lab series. The good internal reliability of both the self-assessment survey and the feedback model further support the utility of EPAs as a reliable tool for feedback in skills lab education. The significant difference between self-assessment and feedback scores indicates the value of EPAs in promoting self-awareness and providing meaningful feedback.
Farris et al., 2023 [14]; United States of America (USA)	To evaluate pharmacy students' self-identified levels of entrustability before and after their advanced pharmacy practice experiences (APPE).	Practice experiences (APPE) in different settings	15 EPAs from the Core Entrustable Professional Activities for new pharmacy graduates in the USA (2016 version)	Not informed	Pharmacy students increased their self-reported level of entrustability over all 15 EPA domains from pre-APPE to post-APPE year.

Table S1. Data from studies included in the review (continued)

Authors; Year; Country	Main objectives	Type of educational activity	Characteristics of EPAs	Use of EPAs in teaching and assessment of student performance	Main findings of the study
Fuller *et al,* 2023 [15]; United States of America (USA)	The purpose of this study was to determine pharmacy preceptor perceptions of an EPA evaluation tool redesigned in 2019 and launched for use in May 2020	Practice experiences (3 IPPEs and 9 APPEs) in the following settings: community pharmacy, health system, ambulatory care, general medicine, inpatient clinical	A list of EPAs developed for early pharmacy practice experiences of the pharmacy program at the University of North Carolina Eshelman School of Pharmacy	Preceptors observe student performance, provide feedback, and fill a structured form at 2,4, and 6 weeks for IPPEs at 2 weeks for APPEs. Summative evaluations are completed at the end of each experience. Before the redesign of the practice experiences, a student's grade was based solely on their ability to obtain or exceed the suggested level of entrustment for each EPA. To minimize the risk of preceptors modifying their prior EPA assessments, the assessment was changed to allow for formative feedback on EPA performance to be separated from the final grade determination. In the holistic grading approach of the new evaluation tool, preceptors are provided with narratives (vignettes) for competency domains and tasked with scoring each domain as either meeting or not meeting performance expectations.	Overarchingly, preceptors perceived that the new EPA assessment tool allowed them to provide feedback and grades more accurately, efficiently, effectively, and easily
Beckett et al, 2023 [16]; United States of America (USA)	To document the performance of first-year pharmacy students on a revised objective structured clinical examination (OSCE) based on national entrustable professional activities, identify risk factors for poor performance, and assess its validity and reliability	A series of OSCEs within the professional pharmacy program	Fourteen EPAs from the Core Entrustable Professional Activities for new pharmacy graduates in the USA (2016 version)	Faculty members observe the student performance, fill a structured form, and provide feedback and grades to the students. Each EPA could have multiple assessed elements. In OSCE-1, there was required a level of entrustment of L1 (ready for thoughtful observation), and for subsequent a level of L2 (moderate level of entrustment, ready for proactive supervision).	A newly revised OSCE was developed using backward design based on best practices and centered on assessment of the national EPAs. Although predictors of poor performance were not identified when accounting for covariates, the OSCE was found to have good validity and reliability.

Table S1. Data from studies included in the review (continued)

Authors; Year; Country	Main objectives	Type of educational activity	Characteristics of EPAs	Use of EPAs in teaching and assessment of student performance	Main findings of the study
Nasser *et al,* 2024 [17]; Lebanon	The aim of this study was to describe the development and implementation of a pharmacy OSCE with a focus on EPAs and Ability Statements (AS), and report the student performance on competencies related to APPE- and practice-readiness such as ethical and legal behaviors, general communication skills, and interprofessional collaboration	OSCEs within the professional pharmacy program. There were 3 different OSCE stations: station 1 for best possible medication history; station 2 for patient education; and station 3 for healthcare provider (HCP) communication	Eight EPAs from the Core Entrustable Professional Activities for new pharmacy graduates in the USA (2016 version)	In each OSCE station, assessment of student performance on EPAs was performed by a faculty observer and other evaluators, who simulate the role of standardized patient or health care provider.	The evaluation of students' ethical and legal behaviors, the interprofessional general communication, and collaboration showed average scores of 82.6%, 88.3%, 89.3%, respectively. Student performance on communication-related statements exceeded 80% in all 3 stations.

Table S1. Data from studies included in the review (continued)

Authors; Year; Country	Main objectives	Type of educational activity	Characteristics of EPAs	Use of EPAs in teaching and assessment of student performance	Main findings of the study
Shtaynberg et al, 2024 [18]; United States of America (USA)	To outline an approach to help students achieve Entrustable Professional Activities (EPAs) entrustment during a sequence of Advanced Pharmacy Practice Experiences (APPEs) by implementing longitudinal monitoring and individualized intervention and remediation strategies	Practice experiences (APPEs) in community and institutional pharmacy, ambulatory and acute care, and non-direct patient care	Fourteen EPAs from the Core Entrustable Professional Activities for new pharmacy graduates in the USA (2016 version)	Preceptors observe student performance, provide feedback, and fill a structured form. Course syllabi outlined example supporting tasks for each EPA. Professionalism expectations are outlined for students as a separated experiential policy. To pass an APPE, students must minimally achieve a level 3 entrustment (perform reactive supervision) for at least 70% of the course-required EPAs. The faculty performs longitudinal monitoring of student performance on EPAs and implement individualized interventions to address deficiencies, such as elective APPEs, proactive outreach, and simulated activities.	Utilizing a multifaceted strategy provided timely, real-world practice opportunities to improve the students' achievement of EPAs across the APPE curriculum and decreased the need for end-of-year remediation and potential graduation delays

Table S1. Data from studies included in the review (continued)

Authors; Year; Country	Main objectives	Type of educational activity	Characteristics of EPAs	Use of EPAs in teaching and assessment of student performance	Main findings of the study
Elmes-Patel *et al,* 2024 [19]; United States of America (USA)	To explore advanced pharmacy practice experience (APPE) preceptor perspectives including implementation recommendations, barriers, and facilitators to using entrustable professional activity (EPA) assessment in pharmacy experiential education	Practice experiences (APPE) in ambulatory and hospital settings.	Core Entrustable Professional Activities for new pharmacy graduates in the USA (2016 version)	Preceptors observe student performance, provide feedback, and fill a structured form.	EPA-based assessments can be used by preceptors for workplace-based assessments during APPEs. Faculty/preceptor development and student orientation with benchmarking and clear expectations are needed to support implementation.
McDowell *et al*, 2024 [20]; United States of America (USA)	The objective of this study was to evaluate community IPPE preceptors' perceptions regarding the newly implemented EPA-based community IPPE curriculum and corresponding preceptor training	Community pharmacy practice experience (IPPE)	Twelve required and two suggested EPAs from the Core Entrustable Professional Activities for new pharmacy graduates in the USA (2016 version)	Preceptors observe student performance, provide feedback, and fill a structured form	Preceptors' feedback supports the use of an EPA-based community IPPE curriculum to assess student performance and prepare students for community APPEs. Preceptor involvement is valuable in the evaluation of a revised experiential curricula to assure IPPE expectations are appropriate and align with contemporary pharmacy practice.

Table S1. Data from studies included in the review (continued)

Authors; Year; Country	Main objectives	Type of educational activity	Characteristics of EPAs	Use of EPAs in teaching and assessment of student performance	Main findings of the study
Cady *et al,* 2024 [21]; United States of America (USA)	The purpose of this study was to describe the impact of novel, semester-long, interactive ID patient cases on APPE-readiness as assessed by specific EPA ratings (which focused on clinical skills and communication skills) by a national cohort of volunteer pharmacy preceptors	Elective course that utilizes preceptors and faculty to mimic clinician interactions and real-time infectious disease management	Four EPAs from the Core Entrustable Professional Activities for new pharmacy graduates in the USA (2016 version)	Volunteer preceptors and faculty members assess student performance on EPAs and fill a survey. The survey includes four Likert scale questions asking volunteers to assess student performance, in addition to four questions with free-text response soliciting feedback on the cases (and or case presentations if volunteers were present), and three questions to identify which patient cases they were assigned to assess how much time they spent speaking with students. The assessment is performed for two independent cases completed by groups of students.	Preceptors and faculty members agreed that students met the four EPAs evaluated (agreement was 85–100%). This semester-long elective provided "real-time" experience and feedback for pre-APPE students to enhance APPE readiness and reinforce EPAs.
Fuller et al, 2024 [22]; United States of America (USA)	This study aims to evaluate the impact of redesigning an entrustable professional activities (EPAs) assessment tool on the accuracy of student performance assessment within pharmacy education	Practice experiences (3 IPPEs and 9 APPEs) in the following settings: community pharmacy, health system, ambulatory care, general medicine, and inpatient clinical	A list of EPAs developed for early pharmacy practice experiences of the pharmacy program at the University of North Carolina Eshelman School of Pharmacy	Preceptors observe student performance, provide feedback, and fill a structured form at 2,4, and 6 weeks for IPPEs at 2 weeks for APPEs. Summative evaluations are completed at the end of each experience. Before the redesign of the practice experiences, a student's grade was based solely on their ability to obtain or exceed the suggested level of entrustment for each EPA. To minimize the risk of preceptors modifying their prior EPA assessments, the assessment was changed to allow for the formative feedback on EPA performance to be separated from the final grade determination.	The redesigned EPA assessment tool demonstrated a decrease in grade inflation resulting in more accurate assessments. The tool's focus on holistic grading and narrative descriptors contributed to better alignment between preceptor assessment and school-suggested achievement levels.

Table S1. Data from studies included in the review (continued)

Authors; Year; Country	Main objectives	Type of educational activity	Characteristics of EPAs	Use of EPAs in teaching and assessment of student performance	Main findings of the study
Ohman et al., 2025 [23]; United States of America (USA)	This study aims to measure students' perspectives of the LLPM (layered learning practice model) and assess if students perceived they were more able to participate in the American Association of Colleges of Pharmacy (AACP) 2016 Core Entrustable Professional Activities (EPAs) during rotations utilizing the LLPM	Practice experience (APPEs) in different settings	US Core Entrustable Professional Activities for new pharmacy graduates 2016	Preceptors assess student performance and fill a structured form. Some of the APPEs use the LLPM for student interaction and shadowing. The layered learning practice model (LLPM) is a teaching strategy that utilizes a hierarchy of team members to educate learners. In pharmacy practice, this hierarchy consists of various combinations of a supervising pharmacist (often called the "primary preceptor"), non-supervising pharmacists, pharmacy residents, and students. Pharmacy residents or non-supervising pharmacists are typically responsible for daily precepting of the students and the supervising pharmacist oversees their work. The LLPM aims to maximize the pharmacy team by utilizing various levels of learners to provide expanded clinical patient care services.	Rotations utilizing the LLPM had a limited impact on students' self-assessed ability to participate in Core EPAs. Students' individual and collaborative skills increased regardless of rotation practice model type.
Jarrett *et al.*, 2025 [24]; United States of America (USA)	The objective was to evaluate the growth in pharmacy student performance in entrustable professional activity (EPA) assessments across the experiential curriculum based on preceptor assessments on an entrustment–supervision (ES) scale	Skill lab-based courses and practice experiences (IPPEs and APPEs) in different settings	US Core Entrustable Professional Activities for new pharmacy graduates (version) 2016	In the skill lab-based courses, there is a reflection on EPA utilization at their practice site. In the IPPEs and APPEs, preceptors use an entrustment-supervision (ES) scale (provide students with formative feedback and a summative assessment of their performance for each EPA) at midpoint and end of the practice experience.	This study sought to highlight the longitudinal integration of EPAs into a pharmacy experiential curriculum and how it demonstrates student growth over time. The results documented consistent growth and suggested that this assessment framework can be feasibly implemented.

References

1.Rhodes, L. A.; Marciniak, M. W.; McLaughlin, J.; Melendez, C. R.; Leadon, K. I.; Pinelli, N. R. Exploratory Analysis of Entrustable Professional Activities as a Performance Measure During Early Pharmacy Practice Experiences. *Am J Pharm Educ*., **2019**, *83*(2), 6517. https://doi.org/10.5688/ajpe6517

2. Pittenger, A. L.; Gleason, B. L.; Haines, S. T.; Neely, S.; Medina, M. S. Pharmacy Student Perceptions of the Entrustable Professional Activities. *Am J Pharm Educ*. **2019**, *83*(9), 7274. https://doi.org/10.5688/ajpe7274.

3. Lounsbery, J. L.; Von Hoff, B. A.; Chapman, S. A.; Frail, C. K.; Moon, J. Y.; Philbrick, A. M.; Rivers, Z.; Pereira, C. Tracked Patient Encounters During Advanced Pharmacy Practice Experiences and Skill Self-assessment Using Entrustable Professional Activities. *Am J Pharm Educ*. **2019**, *83*(9), 7349. https://doi.org/10.5688/ajpe7349.

4. Rivkin, A.; Rozaklis, L.; Falbaum, S. A Program to Prepare Clinical Pharmacy Faculty Members to Use Entrustable Professional Activities in Experiential Education. *Am J Pharm Educ*. **2020**, *84*(9), ajpe7897. https://doi.org/10.5688/ajpe7897

5. Smith, C.; Stewart, R.; Smith, G.; Anderson, H. G.; Baggarly, S. Developing and Implementing an Entrustable Professional Activity Assessment for Pharmacy Practice Experiences. *Am J Pharm Educ*. **2020,** *84*(9), ajpe7876. https://doi.org/10.5688/ajpe7876.

6. Croft, H.; Gilligan, C.; Rasiah, R.; Levett-Jones, T.; Schneider, J. Development and inclusion of an entrustable professional activity (EPA) scale in a simulation-based medicine dispensing assessment. *Curr Pharm Teach Learn*. **2020,** *12*(2), 203–212. https://doi.org/10.1016/j.cptl.2019.11.015.

7. Marshall, L. L.; Kinsey, J.; Nykamp, D.; Momary, K. Evaluating Practice Readiness of Advanced Pharmacy Practice Experience Students Using the Core Entrustable Professional Activities. *Am J Pharm Educ*. **2020,** *84*(10), ajpe7853. https://doi.org/10.5688/ajpe7853.

8. Law, M.; Drame, I.; McKoy-Beach, Y.; Adesina, S. A Six-Semester Integrated Pharmacy Practice Course Based on Entrustable Professional Activities. *Am J Pharm Educ*. **2021**, *85*(1), 848017. https://doi.org/10.5688/ajpe848017

9. Borja-Hart, N. L.; Rowe, A. S.; Gatwood, J.,; Wheeler, J. Incorporation of a mock pharmacy and therapeutics committee as an entrustable professional activity supporting task. *Curr Pharm Teach Learn*. **2021,** *13*(7), 784–788. https://doi.org/10.1016/j.cptl.2021.03.010

10. Sjoquist, L. K.; Bush, A. A.; Marciniak, M. W.; Pinelli, N. R. An Exploration of Preceptor-Provided Written Feedback on Entrustable Professional Activities During Early Practice Experiences. *Am J Pharm Educ*. **2021,** *85*(3), 8091. https://doi.org/10.5688/ajpe8091.

11. Rathbone, A. P.; Richardson, C. L.; Mundell, A.; Lau, W. M.; Nazar, H. Exploring the role of pharmacy students using entrustable professional activities to complete medication histories and deliver patient counselling services in secondary care. *Explor. Res. Clin. Soc. Pharm,* **2021**, *4*, 100079. https://doi.org/10.1016/j.rcsop.2021.100079.

12. Eudaley, S. T.; Brooks, S. P.; Jones, M. J.; Franks, A. S.; Dabbs, W. S.; Chamberlin, S. M. Evaluation of student-perceived growth in entrustable professional activities after involvement in a transitions-of-care process within an adult medicine advanced pharmacy practice experience. *Curr Pharm Teach Learn*. **2022,** *14*(2), 193–199. https://doi.org/10.1016/j.cptl.2021.11.032.

13. Schroeder, M.N.; Murphy, J.A.; Lengel, A.J.; Cruz, B.D. A level of trust: Exploring entrustable professional activities as a feedback tool in a skills lab. *Curr Pharm Teach Learn*. **2023**, *15*, 1034–1039. https://doi.org/10.1016/j.cptl.2023.10.007

14. Farris, C.; Fowler, M.; Wang, S.; Wong, E.; & Ivy, D. Descriptive survey of pharmacy students' self-evaluation of Advanced Pharmacy Practice Experiences (APPE) and practice readiness using entrustable professional activities. *Pharm Educ*, **2023**, *23*(1), p. 447–453. https://doi.org/10.46542/pe.2023.231.447453.

15. Fuller, K.; Crescenzi, A.; Pinelli, N. R. Preceptor perceptions of a redesigned entrustable professional activity (EPA) assessment tool in pharmacy practice experiences. *Curr Pharm Teach Learn*. **2023,** *15*(7), 666–672. https://doi.org/10.1016/j.cptl.2023.06.018.

16. Beckett, R. D.; Gratz, M. A.; Marwitz, K. K.; Hanson, K. M.; Isch, J.; Robison, H. D. Development, Validation, and Reliability of a P1 Objective Structured Clinical Examination Assessing the National EPAs. *Am J Pharm Educ*. **2023**, *87*(6), 100054. https://doi.org/10.1016/j.ajpe.2023.100054

17. Nasser, S. C.; Kanbar, R.; Btaiche, I. F.; Mansour, H.; Elkhoury, R.; Aoun, C.; Karaoui, L. R. Entrustable professional activities-based objective structured clinical examinations in a pharmacy curriculum. *BMC med educ*, **2024**, *24*(1), 436. https://doi.org/10.1186/s12909-024-05425-y

18. Shtaynberg, J.; Rivkin, A.; Rozaklis, L.; Gallipani, A. Multifaceted Strategy That Improves Students' Achievement of Entrustable Professional Activities Across Advanced Pharmacy Practice Experiences. *Am J Pharm Educ*. **2024**, *88*(9), 100755. https://doi.org/10.1016/j.ajpe.2024.100755

19. Elmes-Patel, A. T.; Allen, S. M.; Djuric Kachlic, M.; Schriever, A. E.; Driscoll, T. P.; Tekian, A.; Cheung, J. J. H.; Podsiadlik, E.; Haines, S. T.; Schwartz, A.; Jarrett, J. B. Preceptor Perspectives Using Entrustable Professional Activity-Based Assessments During Advanced Pharmacy Practice Experiences. *Am J Pharm Educ*. **2024.** *88*(12), 101332. https://doi.org/10.1016/j.ajpe.2024.101332.

2o. McDowell, L.; Hamrick, J.; Fetterman, J.; Brooks, K. Preceptors' perceptions of an entrustable professional activities-based community introductory pharmacy practice experience curriculum. *Curr Pharm Teach Learn*. **2024,** *16*(2), 109–118. https://doi.org/10.1016/j.cptl.2023.12.026

21. Cady, E. A.; Dillon, A. J.; Bourland, K.; Rybakov, I.; Cluck, D. B.; Veve, M. P. You'll have to call the attending: Impact of a longitudinal, "real-time" case-based infectious diseases elective on entrustable professional activities to enhance APPE readiness. *Curr Pharm Teach Learn., * **2024**, 16(8), 102092. https://doi.org/10.1016/j.cptl.2024.04.008

22. Fuller, K.; Pinelli, N. R.; Persky, A. M. Redesigned Entrustable Professional Activity (EPA) Assessments Reduce Grade Inflation in the Experiential Setting. *Am J Pharm Educ*. **2024,** *88*(11), 101297. https://doi.org/10.1016/j.ajpe.2024.101297

23. Ohman, T. A.; Richter, L. M.; Dewey, M.; Vigen, K. Multisite survey of pharmacy student perspectives of the layered learning model and ability to participate in core entrustable professional activities during advanced pharmacy practice experiences. *Curr Pharm Teach Learn*. **2025**. 17(10), 102436. https://doi.org/10.1016/j.cptl.2025.102436

24. Jarrett, J. B.; Elmes-Patel, A. T.; Allen, S. M.; Djuric Kachlic, M.; Schriever, A. E.; Driscoll, T. P.; Tekian, A.; Cheung, J. J. H.; Podsiadlik, E.; Haines, S. T.; Schwartz, A. Longitudinal Preceptor Assessment of Entrustable Professional Activities Across Introductory and Advanced Pharmacy Practice Experiences. Pharmacy (Basel, Switzerland), **2025**, 13(3), 72. https://doi.org/10.3390/pharmacy13030072
